# Inactive *trans*-Sialidase Expression in *iTS-null Trypanosoma cruzi* Generates Virulent Trypomastigotes

**DOI:** 10.3389/fcimb.2017.00430

**Published:** 2017-10-04

**Authors:** Carla A. Pascuale, Juan M. Burgos, Miriam Postan, Andrés B. Lantos, Adriano Bertelli, Oscar Campetella, M. Susana Leguizamón

**Affiliations:** ^1^Instituto de Investigaciones Biotecnológicas, Universidad Nacional de San Martín, Buenos Aires, Argentina; ^2^Consejo Nacional de Investigaciones Científicas y Técnicas, Buenos Aires, Argentina; ^3^Instituto Nacional de Parasitología “Dr. Mario Fatala Chabén”, Administración Nacional de Laboratorio e Institutos de Salud, “Dr. Carlos G. Malbrán”, Buenos Aires, Argentina

**Keywords:** Discrete Typing Units, inactive *trans*-sialidase, pathogenesis, *Trypanosoma cruzi* virulence, virulence factors

## Abstract

Disclosing virulence factors from pathogens is required to better understand the pathogenic mechanisms involved in their interaction with the host. In the case of *Trypanosoma cruzi* several molecules are associated with virulence. Among them, the *trans*-sialidase (TS) has arisen as one of particular relevance due to its effect on the immune system and involvement in the interaction/invasion of the host cells. The presence of conserved genes encoding for an inactive TS (iTS) isoform is puzzlingly restricted to the genome of parasites from the Discrete Typing Units TcII, TcV, and TcVI, which include highly virulent strains. Previous *in vitro* results using recombinant iTS support that this isoform could play a different or complementary pathogenic role to that of the enzymatically active protein. However, direct evidence involving iTS in *in vivo* pathogenesis and invasion is still lacking. Here we faced this challenge by transfecting *iTS-null* parasites with a recombinant gene that allowed us to follow its expression and association with pathological events. We found that iTS expression improves parasite invasion of host cells and increases their *in vivo* virulence for mice as shown by histopathologic findings in heart and skeletal muscle.

## Introduction

Chagas disease, the American trypanosomiasis, is a chronic, disabling parasitic disease caused by the flagellate protozoon *Trypanosoma cruzi*. Currently, about 6 million people are infected and 13% of Latin America population is at risk of infection, representing a major health, social, and economic problem in Latin America and a global emerging threat due to the population migration (Rassi et al., 2010; WHO-World Health Organization, 2015).

During the acute phase of the infection, *T. cruzi* induces several immune system alterations that allow the parasite to disseminate and persist in the host (Dos Reis, 2011). Several parasite molecules are associated with virulence. Among them the *trans*-sialidase (TS), an enzyme expressed and shed by the trypomastigote stage, has arisen as one of particular relevance due to its effect on the immune system and involvement in the interaction/invasion of the host cell, playing also a central role in the parasite biology (Oliveira et al., 2014). *T. cruzi* is unable to synthesize sialic acids *de novo* and thus, the surface membrane glycoconjugates are sialylated by TS that obtain this sugar from the host glycoconjugates (Mucci et al., 2017). Acquisition of the sialyl residue allows the parasite to survive in blood and disseminate the infection. This virulence factor is also shed and distributed by the bloodstream thanks to a repetitive antigenic *C*-terminus extension known as SAPA (for Shed Acute Phase Antigen) that extends its half-life in blood (Buscaglia et al., 1999; Alvarez et al., 2004). By this way, TS acts systemically inducing several abnormalities in the host's immune system including depletion of thymocytes (Leguizamón et al., 1999; Mucci et al., 2006) and absence of germinal centers in secondary organs (Risso et al., 2007), along with thrombocytopenia and erythropenia (Tribulatti et al., 2005).

A large TS gene family of about 1,430 members has become evident after the release of the *T. cruzi* genome (El-Sayed et al., 2005). TS-homologous proteins are encoded by genes originally distributed in four families (Campetella et al., 1992) now separated in eight groups (Freitas et al., 2011). Only one of these groups contains about a dozen genes that encodes for the active enzyme. This figure is certainly underestimated due to the inevitable collapse during the assembly of almost identical sequences. In fact, the gene group that includes the enzymatically active TS (aTS) was estimated to contain from just a couple to about 150 genes by using different quantification techniques along several *T. cruzi* strains (Cremona et al., 1999; Burgos et al., 2013). These TS-encoding genes are in turn distributed about in halves based in the presence of a T/C transition in the codon that encodes the Tyr_342_, which is crucial in the enzyme activity (Cremona et al., 1995). This mutation, that leads to a Tyr_342_His, is notably conserved as such in all the genes from different parasites strains that encode the so-called enzymatically-inactive proteins (iTS) of this family. However, iTS-encoding genes do not contain other inactivating alterations in their sequence (cumulative mutations, insertions/deletions, stop codons, etc.) being thus not pseudogenes abandoned along the evolution (Cremona et al., 1995; Burgos et al., 2013).

*T. cruzi* population is clonal and includes six main Discrete Typing Units (DTU) known as TcI to TcVI (Zingales et al., 2009). These DTUs are characterized by genetic tags, being the expression of TS a biological marker associated with their differential virulence (Risso et al., 2004). From the TS genes sequences available in data banks, we have designed a collection of primers that allow the amplification and sequencing of the region where the T/C mutation (encoding Tyr_342_His) is included. After analyzing more than 30 parasite isolates from different hosts along the Americas (Burgos et al., 2013) we found that, in contrast to higher-virulence strains (from DTUs TcII, TcV, and TcVI), lower-virulence parasite strains (from DTUs TcI, TcIII, and TcIV), which display reduced pathogenic findings in mice with low mortality, do not have the iTS-encoding genes (Risso et al., 2004; Burgos et al., 2013). It can be therefore postulated that the acquisition of the *iTS* gene during segregation in evolution is associated with parasite virulence (Burgos et al., 2013). Working with a recombinant iTS, we and others (Cremona et al., 1999; Todeschini et al., 2002a) determined an associated lectin-like activity binding to appropriate substrate sugars on the cell surfaces and in solution. Even more, the analysis of this TS isoform by crystallographic studies (Oppezzo et al., 2011) indicates that it hydrolyzes a substrate sugar, although at a much lower rate than its aTS counterpart, which might allow the iTS to bind and release, allowing its recycling. The presence of conserved genes coding for the iTS was always puzzling. The notably conservation of His_342_ along different parasite DTUs, strongly supports a defined biological function that involves the interaction with the sugar substrate. The evolutionary acquisition by virulent parasites of *iTS* is a challenging hypothesis (Burgos et al., 2013). Studies with recombinant iTS allowed to associate this isoform with immune alterations such as costimulation and deviation of the elicited response (Todeschini et al., 2002b; Ruiz Díaz et al., 2015). Recently, we have reported that iTS/aTS participate in the manipulation of the T CD4 cells all along their maturation stages favoring the elicitation of a TH2 response (Ruiz Díaz et al., 2015). However, the actual relevance of iTS in the pathogenesis of Chagas disease remains elusive since direct evidence involving iTS in *in vivo* pathogenesis and invasion is still lacking.

Taken together, these findings suggest that iTS proteins could have alternative or complementary roles to the active TS in parasite virulence for mammals. However, due to the inherent difficulties to distinguish the expression of two proteins that essentially differ in a single aminoacid residue, this hypothesis has not yet been addressed. Taking advantage of the absence of *iTS* genes in the TcI low-virulence parasites (Burgos et al., 2013), here we faced this challenge by transfecting *iTS-null* parasites with a recombinant *iTS* gene that allowed us to follow its expression and association with pathological events.

## Materials and methods

### Animals

This study was carried out in accordance with the recommendation of the Committee on the Ethics of Animal Experiments of the Universidad Nacional de San Martín (UNSAM), following the recommendations of the Guide for the Care and Use of Laboratory Animals of the National Institutes of Health. The protocol was approved by the Institutional Committee on the Ethics of Animal Experiments of the UNSAM (CICUAE). C3H/HeJ and BALB/cJ mice were obtained from The Jackson Labs and CF1 from Charles River and bred in our facilities. Male mice (60–90 days old) were used in all experiments. Sprague Dawley suckling rats were obtained from the Animal Facility of the Facultad de Veterinaria, Universidad de Buenos Aires.

### Parasite culture

Epimastigote forms of the K98 clone, derived from *T. cruzi* CA-I strain (Gonzalez Cappa et al., 1999), DTU TcI lacking of *iTS* gene (Burgos et al., 2013), were grown at 28°C in liver infusion tryptose (LIT) medium supplemented with 10% fetal bovine serum (FBS) as described by Camargo (1964). Mammalian stages were grown in Vero cell cultures by infecting cells with metacyclic trypomastigote derived from epimastigote cultures (Zingales and Colli, 1985).

### DNA and plasmid constructions

**Figure 1:**
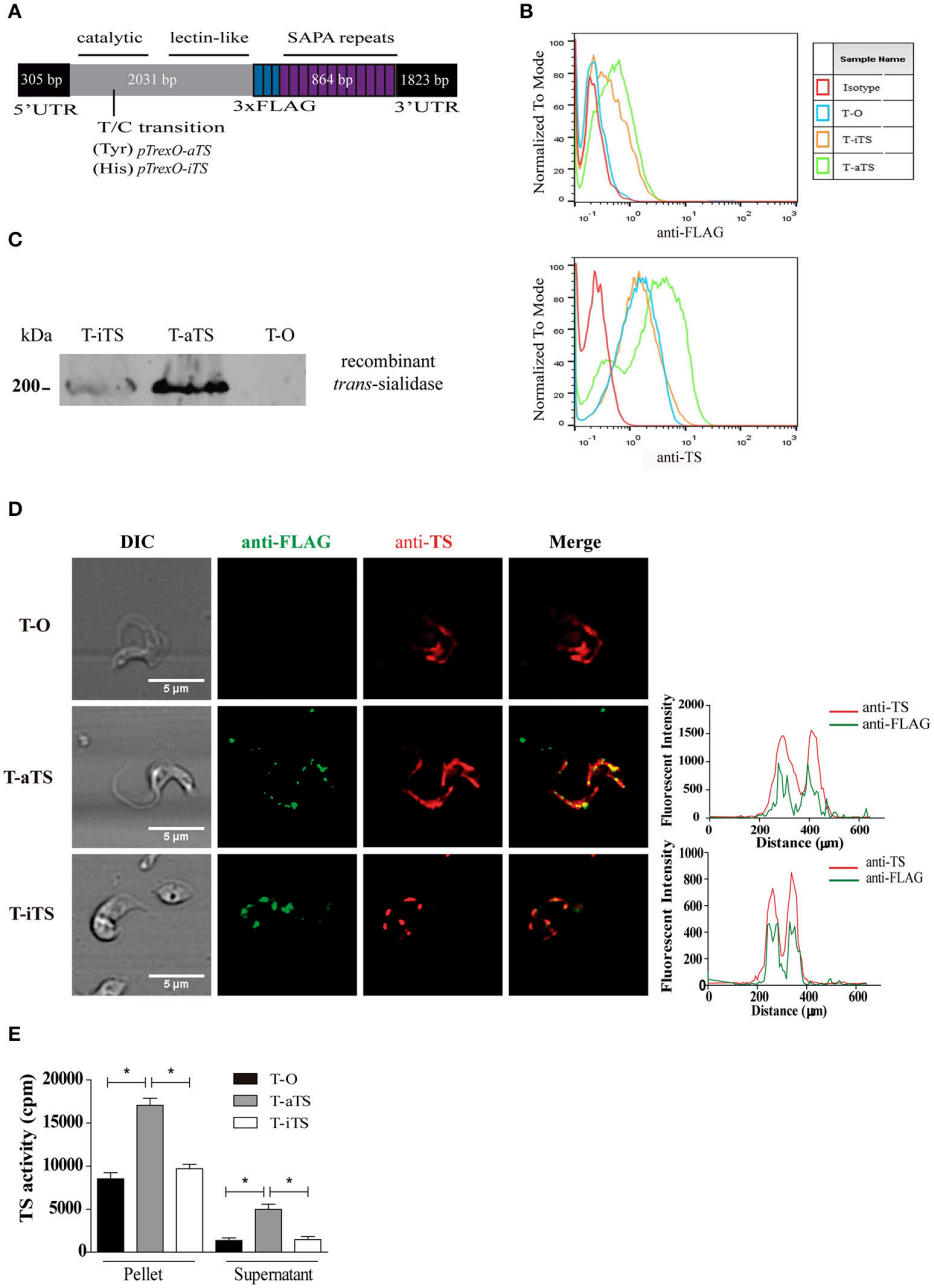
Expression of iTS and aTS recombinant proteins. **(A)** Schematic representation of *iTS* and *aTS* recombinant genes. The TSs with an inserted 3XFLAG epitope-encoding sequences were cloned together with the 3′ and 5′ UTRs into the *pTrex-Omni* vector. The location of the T/C transition encoding for Tyr (aTS) or His (iTS) respectively, is shown. For further details, see GenBank accession numbers MF405919 and MF405920. **(B)** Flow cytometry analysis of TSs parasite expression. TS expression on T-O, T-iTS, and T-aTS parasites was followed with anti-FLAG antibody (Upper panel) and mAb 13G9 (Lower panel*)*. Isotype control, T-O parasites, were treated with secondary antibodies only. **(C)** Expression of recombinant TSs by Western blot. Anti-FLAG reactivity was only observed in T-iTS and T-aTS trypomastigotes. **(D)** Surface expression pattern of TSs. Confocal images show a similar surface expression pattern of TSs (observed by mAb 13G9 in red) and recombinant TSs (anti-FLAG in green). Fluorescence intensity profiles shows overlapping signals. **(E)** *Ex vivo* expression of *trans-*sialidase activity. Both supernatant and extracts, obtained after trypomastigotes shedding, showed that T-aTS express higher activity respect to T-iTS and T-O parasites (*p* < 0.05). The level of TS expression in T-iTS and T-O were similar. Results are expressed as mean ± SEM of the replicate values of three independent assays ^*^p < 0.05.

Recombinant genes encoding for iTS and aTS, including twenty-four SAPA repeats and a 3XFLAG epitope together with the 5′UTR and 3′UTR were engineered from previously reported genes sequences (Cremona et al., 1995; Jäger et al., 2008). Regions of interest were amplified by PCR and synthetic DNA encoding the 3XFLAG epitope was inserted and assembled in *pGEMT-Easy* (see scheme in Figure 1A). The full constructs were cloned into the *EcoR*V-*Sal*I sites of the *T. cruzi* expression vector *pTrex-Omni*, kindly provided by Dr. Claudio Pereira (Cámara et al., 2013). The final constructs were fully sequenced. For full sequences see GenBank accession numbers MF405919 and MF405920.

### Parasite transfection

Transfections were carried out with a BTX 600 electroporator in 2-mm gap cuvettes. Epimastigotes (300 × 10^6^) were harvested and washed with LIT medium, resuspended in 0.4 ml of LIT with 100 μg supercoiled plasmid DNA. The electroporation setting was: 1,500 μF, 335 V, and 24 Ω. Parasites were recovered in 5 ml of LIT supplemented with 10% FBS and 48 h later G418 (Sigma) was added at a final concentration of 500 μg/ml. The *neo* resistance gene was used for selection and as an internal control of transfection levels since it is transcribed polycistronically from the same promoter (Di Noia, 2000).

### *In vitro* parasite differentiation and cloning

Metacyclic trypomastigote were obtained from epimastigote cultures by nutritional stress as described (De Lima et al., 2008). After differentiation from epimastigotes, K98 trypomastigotes transfected with *pTrexO* (vector empty, T-TO), *pTrexO-iTS* (T-iTS), and *pTrexO-aTS* (T-aTS) were obtained. Trypomastigotes were cloned by limiting dilution in Vero cell cultures. Then, parasites were cloned by micromanipulation (NT 88 V3, Nikon Narishige, Japan) under observation in an inverted microscope (Nikon Eclipse TE-300, Nikon, Japan). Vero cell cultures were then infected with a single parasite/well.

### Protein detection

Western blots were performed using total *T. cruzi* extracts fractionated by electrophoresis in polyacrylamide denaturing gels and transferred to polyvinylidene fluoride membranes. They were treated for 1 h with 2.5% BSA in PBS and then incubated with the primary antibody overnight, using rabbit IgG anti-FLAG diluted 1:300 (Sigma). Membranes were then washed and incubated with IRDye 800CW-labeled goat antibodies anti-rabbit IgG (Licor Biosciences) (1/10,000). An Odyssey clx infrared imaging system was used to analyze them.

### Confocal microscopy

Expression of iTS and aTS recombinant proteins by trypomastigotes were analyzed by confocal microscopy. Approximately 10^6^ parasites were washed with PBS supplemented with 1% BSA. Parasites were incubated with rabbit IgG anti-FLAG (Sigma, diluted 1:300) to detect recombinant proteins and the mouse monoclonal antibody (mAb) 13G9 anti-TS (1:100) (Buschiazzo et al., 2002), to detect all *trans*-sialidases either endogenous or recombinant. Parasites were labeled with secondary AlexaFluor568-antibodies anti-rabbit IgG and AlexaFluor488-anti-mouse IgG (diluted 1:1,000, both from Biolegend). Fluorescence imaging was carried out under an Olympus FV1000 microscope equipped with a Plan APO N 60x oil, 1.42 NA, FN 26.5 objective.

### Flow cytometry

Approximately 10^6^ transfected trypomastigotes were washed with PBS supplemented with 1% FBS and incubated with rabbit antibodies anti-FLAG (Sigma) and with mAb 13G9. Then, parasites were labeled with AlexaFluor488-antibodies anti-rabbit IgG and Phycoerythrin (PE)-anti-mouse IgG (both from Biolegend, at 1:500 dilution). Parasites were then fixed with 4% paraformaldehyde in PBS and fluorescence was analyzed in a CyFlow space cytometer (Partec, Germany).

### TS activity in *in vitro* assays

Enzymatic activity was assayed in parasites and their supernatants after allowing them to shed for 1 h at 37°C in PBS-BSA. The transference of the sialyl residue from 3′sialyl-lactose to [^14^C]-lactose was performed as described earlier (Leguizamón et al., 1994).

### Recombinant iTS production and purification

Recombinant iTS (riTS) (Cremona et al., 1995) was expressed in *Escherichia coli* BL21 and purified to homogeneity by immobilized metal affinity chromatography through Ni^2+^-charged Hi-Trap chelating columns (GE Healthcare) and ion-exchange chromatography (Mono Q; GE Healthcare) as described previously (Leguizamón et al., 1999).

### Vero cells infection

Approximately 10^4^ Vero cells were plated in 24-well plates containing round glass coverslips with DMEM (Gibco) supplemented with 10% FBS and penicillin/streptomycin. Vero cell infection was allowed for 1 or 18 h at 37°C at a multiplicity of infection (moi) of 1:1, using cell culture-derived trypomastigotes from *T. cruzi* transfected clones. After infection, cultures were washed with PBS to eliminate free parasites and re-incubated with fresh medium for 3 days before fixation with 4% paraformaldehyde in PBS. Fixed cultures were stained with DAPI, and the coverslips mounted onto glass slides with Fluorsave (Calbiochem) for microscopic analysis. The number of infected cells and the number of amastigotes per cell were determined by counting 500 Vero cells per coverslip for each group. Three independent experiments were performed for each infection.

#### Infection with recombinant iTS preincubation assays

Vero cells were incubated with riTS (5, 15, and 50 μg/ml) during 30 min. Then, cells were washed twice with PBS before starting *T. cruzi* infection during 1 h at 37°C (moi 1:10), using cell culture-derived trypomastigotes from *T. cruzi* transfected clones. Three days after, fixing, staining and analysis were carried out as described above. In other set of assays, *T. cruzi* infection was carried out in riTS presence (50 μg/ml) but without the washing steps before parasite addition. Vero infection were analyzed as above.

### Primary cardiocytes and cardiac fibroblasts isolation

Hearts were obtained from Sprague Dawley rats (3–5 days old), and washed twice in PBS-1% glucose. Then minced and digested in trypsin 0.16%, collagenase 0.016%, and DNase I 0.002% for 8 min for 6–7 times at 37°C with periodic mixing. Digests were pooled, stopped with cold PBS containing glucose 1%, FBS 4%, and DNAse I 0.004%. Supernatant was then transferred to a 15 milliliters centrifuge tube and centrifuged at 4°C, 800 G for 10 min. The pellet was collected and washed in DMEM/F12 (Gibco) with 10% FBS and penicillin/streptomycin. Cells were incubated at 10^6^ cell/well in six-well plates. After 3 h, non-adherent cells (cardiomyocytes) were removed and plated onto 24-well plates containing glass coverslips and DMEM/F12 supplemented with 10% FBS (Gibco) and antibiotics. The adherent cells (mainly cardiac fibroblasts) were grown in DMEM with 10% FBS and antibiotics (Wallukat and Wollenberger, 1987). Cells were infected at a moi 1:1 cell/parasite, using 10^5^ cells. The interaction time was 1 or 18 h.

### Macrophages infection

Macrophages were harvested from the peritoneal cavity of C3H/HeJ and BALB/cJ naive mice. Cells were harvested with cold RPMI 1640 (Gibco). Peritoneal macrophages were plated on coverslips in 24-well plates (10^5^ cells/well). After 1 h at 37°C in 5% CO_2_, adherent cells were washed with PBS and cultured in DMEM supplemented with 5% FBS plus penicillin/streptomycin. Peritoneal macrophages were infected at a moi 1:1 cell/parasite for 18 h following the same protocol as above.

### Mice infection

Groups of five CF1 male mice (15 days old) were inoculated intraperitoneally with 5 × 10^4^ Vero cell culture-derived trypomastigotes. Then, bloodstream parasites were used to infect twelve BALB/cJ male mice (60 days old) per group with 5 × 10^4^ bloodstream trypomastigotes. Mice were observed daily to determine mortality and morbidity. Circulating parasites were monitored starting at 11 days post-infection (dpi) and parasitaemia was evaluated using a Neubauer hemocytometer (Risso et al., 2004).

### Histopathology

Heart and skeletal muscle samples were collected from non-infected and infected mice, fixed in 4% paraformaldehyde and embedded in paraffin. Five-micron sections were codified, stained with hematoxylin-eosin, and evaluated under light microscope as described elsewhere (Postan et al., 1983; Martin et al., 2007). Briefly, inflammation rates in cardiac samples (auricles and ventricles) and skeletal muscle sections were graded from 0 to 4 for normal, focal or a single inflammatory focus, multifocal but non-confluent inflammatory infiltrates, confluent inflammation with partial section involvement, and diffuse inflammation extended through the section, respectively. For each heart sample an inflammation score was taken as an average of all analyzed regions. All slides were evaluated independently by researchers who were blinded to the sample status.

### Statistical analysis

Data were expressed as means ± standard errors. The Student *t-*test was used to analyze the statistical significance of assays. The Log-rank (Mantel Cox) test was used to compare survival curves. Presence of amastigotes nest among infected groups was analyzed by Fisher exact test. Differences were considered significant at *p* < 0.05. All analyses were performed with Prism software (version 5.0; GraphPad).

## Results

### iTS recombinant proteins expression in transfected parasites

In order to obtain *T. cruzi* expressing the inactive *trans*-sialidase protein (iTS) low virulence K98 parasites, naturally lacking of *iTS* genes (Burgos et al., 2013), were used. Parasites were genetically modified by transfection with a recombinant *iTS* gene cloned into *pTrexO* expression vector (see scheme in Figure 1A). K98 parasites transfected with the empty vector or carrying the recombinant *aTS* gene (enzymatically active *trans*-sialidase-encoding gen) were used as experimental controls. Both constructs (*pTrexO-aTS* and *pTrexO-iTS*) also contain the 5′ and 3′ UTRs of the wild type *TS* genes (Jäger et al., 2008) required to drive their expression into the trypomastigote stage. Constructions also contain the sequences encoding signal that lead proteins to the plasmatic membrane, 24 repeats of the SAPA antigen and a 3XFLAG epitope added to distinguish recombinant proteins from those native ones. Epimastigotes of the K98 isolate were transfected by electroporation with the constructs (*pTrexO-iTS, pTrexO-aTS*, or empty *pTrexO*) and selected by G418. Parasites were then differentiated to the metacyclic stage that was used to infect Vero cell cultures. Trypomastigotes from Vero cells were later cloned, thus obtaining parasites expressing iTS or aTS recombinant proteins or none of them (named T-iTS, T-aTS, and T-O parasite clones, respectively).

Expression of TSs proteins were analyzed by means of the mAb 13G9 (anti-TS), which is directed to the catalytic site and recognize both TS isoforms (Buschiazzo et al., 2002), and with an anti-FLAG antibody to identify recombinant TSs. As expected, flow cytometry analysis showed that all trypomastigote groups were labeled by the mAb 13G9 but only T-iTS and T-aTS parasites were reactive with anti-FLAG antibody (Figure 1B). Interestingly, the total amount of TSs, endogenous plus recombinant, was higher in the aTS than in iTS or T-O parasites, which were similar between them (Figure 1B lower panel). The increased total content of TSs (Figure 1B lower panel) and the lower reactivity to iTS (Figure 1B upper panel) correlated with the increased aTS expression found by Western blots of trypomastigote lysates, where the expected ~200 kDa protein is recognized by the anti-FLAG antibodies (Figure 1C). The appropriate localization of recombinant proteins was analyzed by confocal microscopy. Whereas, T-O parasites were reactive only with the mAb 13G9, T-iTS, and T-aTS parasites were reactive for both antibodies (mAb 13G9 and anti-FLAG) and displayed similar fluorescence patterns, as expected (Figure 1D). The fluorescence intensity profile of anti-FLAG and mAb 13G9 antibodies shows the overlap of both signals along the parasite surface (Figure 1D). A colocalization analysis using the Fiji software (Plug-in: Intensity Correlation Analysis, ICA) was carried out, where the Pearson (R) coefficients were R = 0.8964; SEM ± 0.063 and 0.9683 ± 0.063 to T-iTS and T-aTS, respectively. Therefore, the endogenous and recombinant TSs were localized together following the described spotted pattern along the trypomastigote surface (Lantos et al., 2016).

To test the TS enzymatic activity, culture-derived trypomastigotes were pelleted, washed and allowed to shed. T-O and T-iTS parasites displayed similar TS activity both in pellets and supernatants while, in contrast, higher TS activity (*p* < 0.05) was found for T-aTS parasites in both fractions (Figure 1E). Therefore, increased aTS expression was observed in T-aTS parasites either by antibodies reactivity or enzymatic activity. All results obtained indicate the correct expression and location of the recombinant enzymes in transfected trypomastigotes without altering the endogenous TS.

### Trypomastigotes expressing iTS show higher invasion ability

*T. cruzi* has the ability to infect a wide variety of different mammalian cell types. To fully analyze the infection ability of transfected parasites expressing the iTS isoform, non-phagocytic and phagocytic cells were tested. As non-phagocytic cells, epithelial Vero cells, primary rat fibroblasts, and cardiocytes were used, whereas macrophages were tested as phagocytic cells.

**Figure 2:**
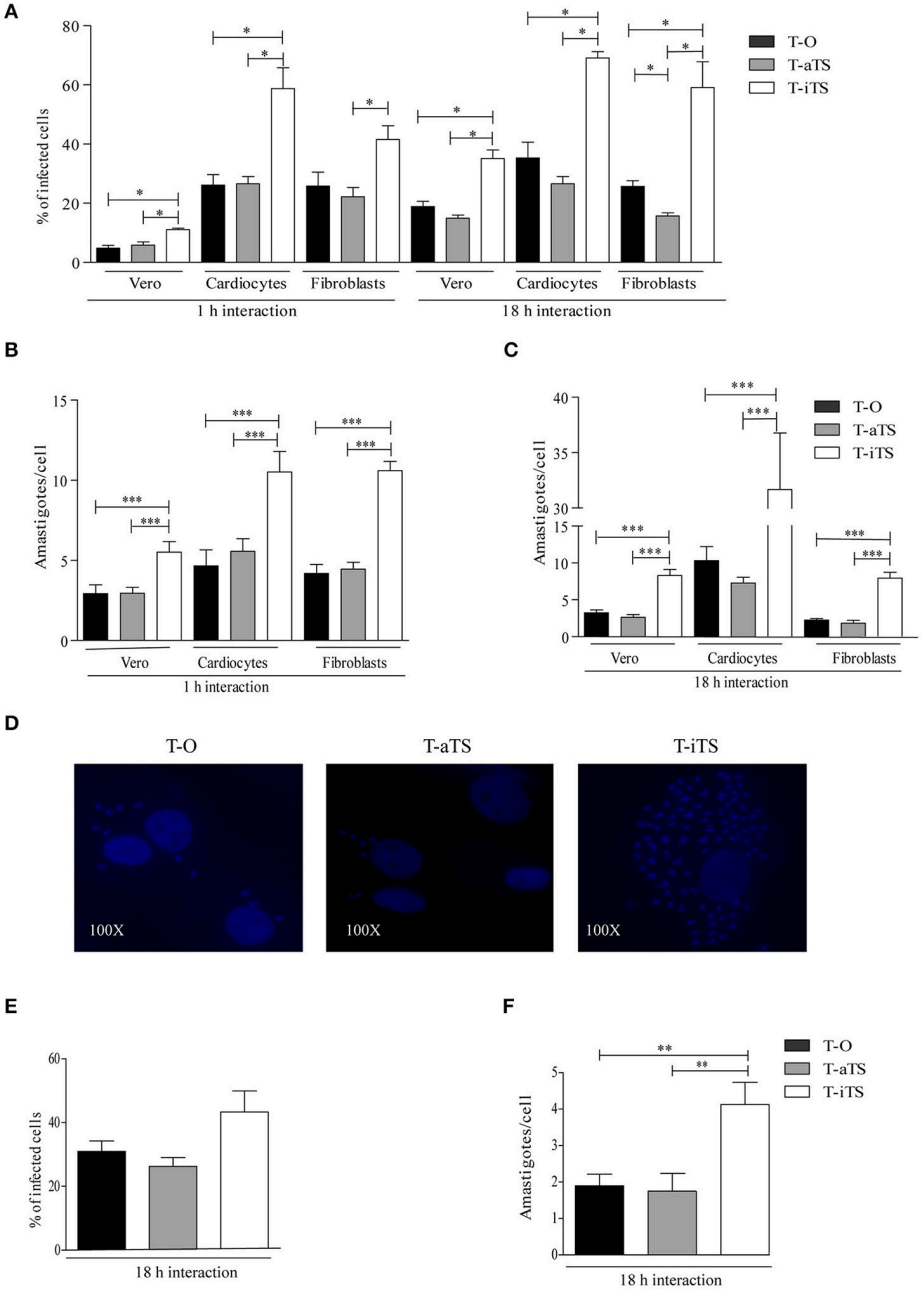
Analysis of iTS role in the infection of non-phagocytic and phagocytic cells. Different assays were carried out with T-O, T-iTS, and T-aTS parasites. **(A)** Infection rate of non-phagocytes cells. Infections (1 and 18 h) were carried out at 1:1 moi. T-iTS parasites were significantly more invasive. For Vero and cardiocytes cells T-iTS parasites infection rates were higher than controls at both measured times (*p* < 0.05). For fibroblast cells the same differences were observed at 18 h of infection (*p* < 0.05). Fibroblasts infection with T-iTS parasites was also superior to T-aTS at 1 h (*p* < 0.05). **(B)** and **(C)** Analysis of the number of amastigotes per cell developed at different interaction times in non-phagocytes cells, T-iTS parasites showed higher number of amastigotes at 1 and 18 h (*p* < 0.001). **(D)** Infection of primary cardiocyte cells. Representative fields of intracellular amastigotes number induced are shown. **(E)** Infection rates in murine macrophages. Similar infection levels were observed at 18 h of interaction. **(F)** Number of amastigotes in murine macrophages. T-iTS showed more amastigotes per cell than controls (*p* < 0.01). Results are expressed as mean ± SEM of the replicate values of three independent cultures. ^*^p < 0.05, ^**^p < 0.01, and ^***^p < 0.001.

Vero cells were infected with T-iTS, T-aTS, and T-O parasites. The number of infected cells and intracellular amastigotes was determined after 18 h of infection. Notably, T-iTS showed higher values of infection (*p* < 0.05) and number of amastigotes/cell (*p* < 0.001) as compared with control T-O and T-aTS (Figures 2A,B). When the parasite-cell interaction lapse was reduced to 1 h (maintaining 1:1 moi), the number of infected cells decreased (*p* < 0.05) although remaining higher than controls, further supporting that these parasites were more infective (Figure 2A). The number of amastigotes/cell was also higher in cells infected by T-iTS than in control groups for all the conditions assayed (Figures 2B,C). Therefore, the increment in the observed infection capacity was independent of the parasite-host cell interaction time.

Primary rat fibroblast cultures were infected as above. At both times of interaction with the different parasites, the infection values were also higher for T-iTS than those obtained with T-aTS (Figure 2A). Moreover, the T-iTS infection rate at 18 h was higher than for T-O parasites *p* < 0.05 (Figure 2A). Strikingly, the intracellular parasite number was also superior in those cultures infected with T-iTS respect to both control groups at 1 and 18 h (Figures 2B,C).

Cardiocytes infection is of particular relevance in the context of further pathology, since inflammation and subsequent fibrosis, induced by parasitic persistence, are responsible for the development of chagasic heart disease (Machado et al., 2013). Cardiocytes obtained from neonatal rats were infected with the different parasite clones. At both interaction times, levels of T-iTS infection were significantly higher (*p* < 0.05) than those observed for T-O, or T-aTS parasites. The number of amastigotes was also higher (*p* < 0.001) in cells infected with T-iTS, relative to T-aTS and T-O group (Figures 2B,C). It is important to note the striking difference observed between the number of amastigotes present in T-iTS infected cardiocytes relative to Vero or primary fibroblast cell cultures at 18 h (Figures 2C,D).

In order to analyze the behavior of T-iTS parasites in phagocytes, murine peritoneal macrophages were used. Infection rates produced by T-iTS parasites were similar to T-aTS and T-O trypomastigotes (Figure 2E). However, the number of amastigotes/cell was again superior in those cells infected by T-iTS than in T-O or T-aTS-infected cultures, *p* < 0.001 (Figure 2F).

#### iTS pre-incubation did not modified invasion of trypomastigotes expressing iTS

**Figure 3:**
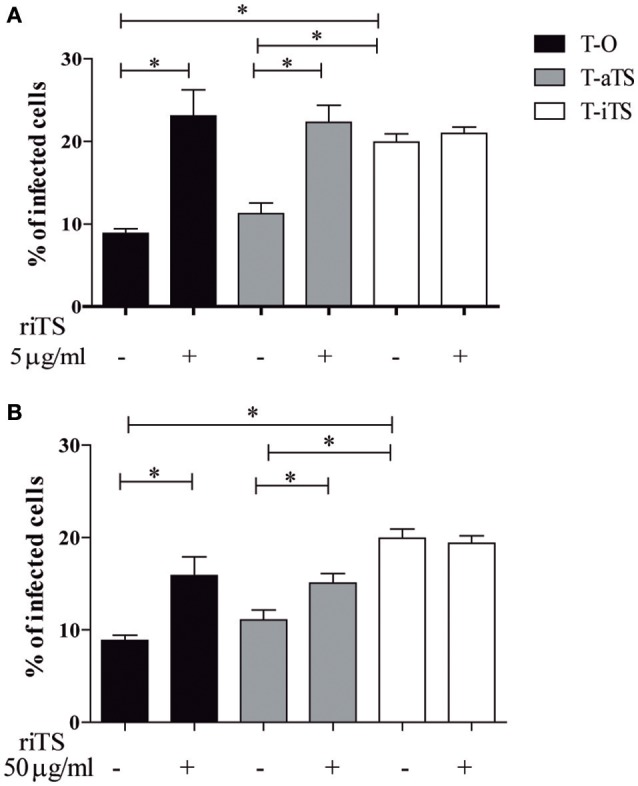
Infection rate of Vero cells in presence of recombinant iTS. Vero cells were treated with riTS before **(A)** or during **(B)** the infection. **(A)** Cells were incubated with 5 μg/ml of riTS during 30 min, then washed and infected with T-O, T-aTS, or T-iTS parasites. **(B)** Cells were preincubated with 50 μg/ml riTS and then infected without washing riTS. Both treatments increased the infection rate in T-O as well as in T-aTS assays (*p* < 0.05) but no difference was observed for T-iTS parasites. Results are expressed as mean ± SEM of the replicate values of three independent cultures. ^*^p < 0.05.

In order to analyze the *in vitro* biological effect of iTS on cell invasion, Vero cells were pre-treated with recombinant iTS (riTS) before infection. Two different protocols were followed. First a pre-treatment of cells with 5, 15, or 50 μg/ml of purified riTS during 30 min was made. After washings, cells were infected with the different parasite clones (T-O, T-iTS, T-aTS) during 1 h. Another assay consisting of treatment with 50 μg/ml of purified riTS during 30 min followed by the infection during 1 h, without washing the remnant recombinant protein was followed. As shown in Figure 3A, previous incubation of Vero cells with riTS (5 μg/ml), induced significant increment of invasion by T-O and T-aTS trypomastigotes, while the invasion values by T-iTS parasites were not modified. Similar results were obtained when 15 or 50 μg/ml were used (data not shown). Simultaneous incubation of riTS, Vero cells and parasites did not modify these results (Figure 3B).

### Inactive *trans*-sialidase expression in natural *iTS-null* parasites increase its virulence in the murine model

**Figure 4:**
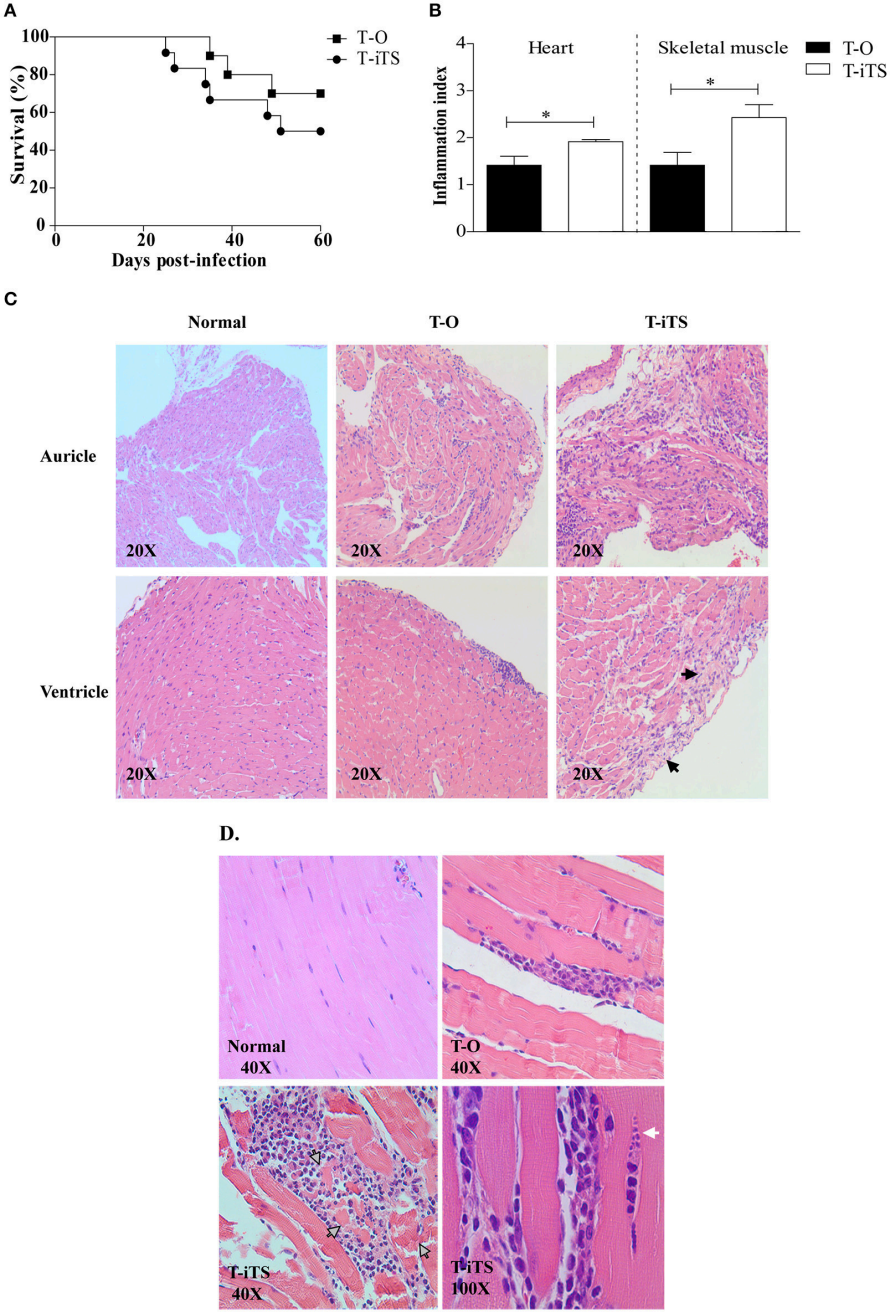
iTS expression increases *Trypanosoma cruzi* virulence in mice. *In vivo* study of transfected parasites virulence. **(A)** Survival rate of T-O and T-iTS infected mice observed during 60 days of infection. **(B)** Inflammation index among heart and skeletal muscle obtained from T-O and T-iTS infected mice. Higher inflammation scores were observed for T-iTS infected mice in both tissue samples (*p* < 0.05). Results are expressed as mean ± SEM of inflammation index among 6 T-iTS and 6 T-O infected mice ^*^p < 0.05. Microphotographs representative of heart **(C)** and skeletal muscle **(D)** histopathological analysis for normal, T-O and T-iTS infected mice. For T-iTS samples notice the presence of necrotic myocardio fibers and inflammatory infiltration (black head arrow), the necrotic fibers and muscle cells remainder (gray head arrow), and a nest of amastigote cells (white head arrow).

Taken into consideration the encouraging results obtained in the *in vitro* infections, we decided to test the T-iTS trypomastigotes pathogenic abilities in the murine host. Male BALB/cJ mice were infected with T-iTS and T-O parasites, parasitaemia, and mortality were recorded during 60 dpi. Mice from both groups showed circulating trypomastigotes but parasitaemia values remained below the minimum level of the quantification method used (10,000 parasites/ml). However, T-iTS-infected mice began to die earlier than control group (25 dpi vs. 35 dpi). Survival rates were 50% (6/12) vs. 72.7% (8/11) for T-iTS and T-O infected mice, respectively (Figure 4A). Skeletal and cardiac muscle samples taken at 60 dpi from surviving mice from both groups were processed for histopathological analysis. Cardiac samples from T-iTS group showed higher inflammation rates than the T-O group (*p* < 0.05; Figure 4B). Necrotic myocardial fibers and higher inflammatory infiltration were observed in T-iTS samples (Figure 4C). Skeletal muscle inflammation degree was also higher in T-iTS infected mice than in T-O ones (*p* < 0.05; Figure 4B). Whereas, T-O mice samples presented multifocal and/or interstitial inflammatory cells infiltration, T-iTS had inflammatory lesions that consisted of necrotic muscle fibers with polymorphonuclears and macrophages infiltration and necrotic parasites (Figure 4D). Interestingly, inflammation rate was accompanied by the presence of amastigote nests in muscle fibers in the T-iTS-infected group (4/6 positive findings) whereas no nests were observed in samples from the control group (0/6 positive findings) (*p* < 0.05; Figure 4D).

Taking together, results obtained from our approach using transgenic parasites allowed us to demonstrate that the iTS isoform is indeed involved in pathogenesis and therefore should be considered as a virulence factor of *T. cruzi*.

## Discussion

To better understand the pathogenic mechanisms involved in *T. cruzi*–host interaction, several virulence factors were studied. The TS, only expressed in trypanosomatides, has been extensively analyzed showing its relevance in invasion process and in the manipulation of host immune system (Oliveira et al., 2014; Freire-de-Lima et al., 2015). Previous *in vitro* results using recombinant iTS support that this isoform could play a different or complementary pathogenic role to that of the enzymatically active protein (Todeschini et al., 2004; Ruiz Díaz et al., 2015). iTS is encoded by conserved genes, that are only present in DTU TcII, TcV, and TcVI, which include highly virulent strains (Burgos et al., 2013). However, direct evidence involving iTS in *in vivo* pathogenesis and invasion was lacking. Here we faced this challenge by transfecting *iTS-null* parasites with a recombinant gene that allowed us to follow its expression and association with pathological events.

The correct expression and localization at the parasite surface of the recombinant TSs were confirmed by several approaches. All parasites assayed reacted with the mAb 13G9 but only TSs-transfected parasites were recognized by the anti-FLAG labeling. Confocal microscopy studies let us observe that the recombinant and the endogenous TSs colocalized and, moreover, they jointly rendered the surface dotted pattern along the trypomastigote surface as previously described (Lantos et al., 2016). These results clearly support that the expression of recombinant TSs does not disturb the normal distribution of the endogenous TS on the parasite surface. Moreover, T-aTS parasites showed more reactivity to anti-FLAG antibodies by cytometry and Western blot assays. In addition, T-aTS parasites produced and shed higher TS activity than T-iTS and T-O parasites. These results indicate that expression of the aTS recombinant protein generate parasites with increased TS activity and preserve their ability to shed these proteins to the milieu. In agreement, the enzymatic activity in T-iTS and T-O parasites did not vary, indicating that the transfection process did not interfere with the endogenous TS expression.

A variety of mammalian cells, either phagocytic or non-phagocytic, are invaded by *T. cruzi* following different mechanisms. The infection process involves events of attachment/detachment along the extracellular matrix until the adhesion and recognition of target cells generate the intracellular signaling involved in the invasion process (De Souza et al., 2010). aTS and iTS have been proposed to participate in these events. aTS through the sialic acid mobilization on host cells and the generation of a sialylated epitope (Schenkman et al., 1991). The iTS seems to be involved through the conserved lectin-like binding properties (Cremona et al., 1999; Todeschini et al., 2004) that might allow the attachment to glycosidic countereceptors (Dias et al., 2008). Here we show that the invasive ability of aTS-transfected parasites was not affected by the increased total TS enzymatic activity induced by the expression of the recombinant aTS. This is in agreement with findings reported by Rubin-de-Celis et al. (2006), who observed that TS activity overexpression does not modify the attachment or invasion parameters. In striking contrast, iTS-transfected parasites increased their invasive ability. The low expression, observed by different techniques, was however enough for the acquired invasion capacity observed not only for Vero cells or primary fibroblast cultures but also for cardiocytes, a parasite preferential target cell. On the other hand, when murine macrophages were infected, no differences were observed among the transfected parasites. A result that might be ascribed to the known *T. cruzi* different invasion processes involved between phagocytic and non-phagocytic cells (De Souza et al., 2010; Caradonna and Burleigh, 2011).

The riTS incubation assays using different concentrations showed the invasion rates increment of T-O and T-aTS but not of T-iTS parasites, either with the pre-treatment or the simultaneous incubation with parasites and Vero cells. These are interesting finding since they are showing, in contrast to iTS lacking parasites, that even low iTS expression is enough to enhance their virulence capacity. Here we also show that 5 μg/ml riTS, induces *T. cruzi* (T-O and T-iTS) invasion increment, in agreement with results reported by Dias et al. (2008) using a pretreatment with 15 μg/ml riTS. Our competition assays results did not differ from that obtained with recombinant protein pre-incubation, suggesting that the interaction of the trypomastigote-expressed iTS with the target cells is mainly relevant in an early step of all the invasion process. An internal signaling pathway might be triggered in the host cell that induce a “permissive state” to the invasion followed, in a second step, by the participation of different surface molecules of the parasite. In any case, our strategy using low virulent *iTS-null* parasites expressing transfected iTS, unequivocally showed the significant increment of invasion of non-phagocytic cells.

Another notable finding with T-iTS parasites was the increased number of amastigotes found in Vero cells, cardiocytes, fibroblasts, and macrophages. This observation was independent of the parasite/cell interaction time allowed (1 or 18 h). It is interesting to note that the number of amastigotes observed in cardiocytes at 18 h of interaction was even higher to that displayed in the other cell types. A plausible explanation for this increment may be provided by the known *T. cruzi* cardiotropism. A more efficient invasion, during the longer time of host cell-parasite contact, may leads to multiple cellular infection that, together with a favored intracellular development, rendered the numerous amastigotes observed. Since the *trans*-sialidase is not expressed in amastigotes and the construction was designed to be expressed only in trypomastigotes, the increased number of amastigotes found does not allows an easy, unique interpretation. Among the several steps in the establishment of an intracellular infection that can be associated with this finding, the generation of parasitophorous vacuoles (PV) containing trypomastigotes and their escape to the cytoplasma is a crucial step (Barrias et al., 2013). The disruption of the PV is associated with the desialylation of the vacuolar membrane due to the sialidase activity of TS (Hall et al., 1992) that, in combination with TcTOX, is the mechanism proposed for *T. cruzi* to access the cytoplasma (Andrews et al., 1990). In support, (Rubin-de-Celis et al., 2006) reported, using Y strain included in Tc II, that trypomastigotes overexpressing TS activity access the cytoplasm faster than control parasites. However, from our findings, it seems that the sole increment of aTS, in trypomastigotes *iTS-null*, is not enough to improve the amastigote number. Therefore, a role for iTS in this process can be postulated, an issue that requires further research. It can be speculated that the observed increment in the amastigote number may be related to higher TS efficiency due to the optimization of the binding of the trypomastigote to the PV membrane mediated by the iTS lectin-like activity.

The most challenging assays for the analysis of iTS as a virulence factor were those developed *in vivo* by mice infection. Mortality of the T-iTS parasite-infected mice began at 25 dpi, that is earlier than T-O infected mice, and reached 50 and 28%, respectively. Even when low parasitaemia levels were observed, histopathological analysis revealed interesting findings. Ventricle and auricle from T-iTS-infected mice hearts showed different and higher degree of inflammation respect those from T-O-infected mice. The relevance of this organ as a preferential *T. cruzi* target is known. Our *in vitro* results showed that T-iTS induces higher infection rates and intracellular amastigotes development. In mice, amastigote nests were not found but the parasite ability to induce alterations in heart tissue was clearly observed (Figure 4C). The development of megacardiopathy, as the main human alteration, is a consequence of parasite persistence inducing inflammation followed by fibrosis, which alter the normal tissue architecture, disturbing organ functioning (Tarleton, 2001). Histological evaluation of skeletal muscle from T-iTS-infected mice also showed increased inflammation index than those infected with T-O parasites. Inflammation values were also superior than that observed in cardiac tissue and was accompanied by the visualization of amastigotes nests in 4/6 T-iTS-infected mice.

As a whole, our results support that the trypomastigote expression of iTS generates more aggressive parasites that not only display increased cell invasion and intracellular amastigotes number, but also the ability to recruit immunological cells, which are in turn involved in increased *T. cruzi*-induced pathogenesis. Therefore, iTS should be considered as another virulence factor from *T. cruzi* whose acquisition during evolution associates with an increment of its pathogenic capacities.

## Author contributions

Conceived and designed the experiments: JB, OC, and ML. Performed the experiments: CP, JB, AL, and AB. Analyzed the data: CP, JB, MP, OC, and ML. Wrote the paper: CP, JB, MP, OC, and ML.

### Conflict of interest statement

The authors declare that the research was conducted in the absence of any commercial or financial relationships that could be construed as a potential conflict of interest.
